# Physicochemical and Multiscale Structural Characterization of Sorghum Cultivars and Their Associations with Anti-Digestion Properties

**DOI:** 10.3390/foods15071127

**Published:** 2026-03-25

**Authors:** Yuan Zhang, Jingjie Lin, Peiyan Li, Danyang Li, Guoyuan Xiong, Kun Yu

**Affiliations:** 1 College of Food Science and Engineering, Anhui Science and Technology University, Chuzhou 233100, China; 2 Anhui Provincial Key Laboratory of Functional Agriculture and Functional Foods, Chuzhou 233100, China; 3 International Joint Research Center of Forage Bio-Breeding in Anhui Province, Chuzhou 233100, China

**Keywords:** sorghum cultivars, anti-digestive properties, sorghum starch structure, functional food ingredients

## Abstract

Sorghum is recognized as a potential functional ingredient with high resistance to digestion. Therefore, this study investigates the anti-digestive properties of eight different types of sorghum cultivars with distinct compositional differences. The results confirmed that the whole sorghum flours exhibit stronger anti-digestive properties compared to its isolated starch, indicating that non-starch components play a role in inhibiting starch digestion. However, there was no significant correlation between the differences in individual components among sorghum varieties and their resistance to digestion. Analysis of sorghum starch structure demonstrated that relative crystallinity and double-helix degree in the long-range ordered architecture show a significant positive correlation with resistant starch (RS). Small-angle X-ray scattering (SAXS) revealed that the relatively thick and dense layered structure of sorghum starch is associated with a lower degree of enzymatic hydrolysis. Gel permeation chromatography (GPC) analysis showed that higher weight-average molecular weight is associated with a higher RS content to a certain extent, while a higher PDI is unfavorable for the formation of digestion-RS structures due to its association with a reduction in the onset gelatinization temperature. Cultivars AH-3, AH-5, and AH-2 with higher molecular weight, narrower molecular weight distribution and denser nanoscale lamellar structures exhibit superior digestion resistance. This research provides a reference for the screening of low-glycemic-index sorghum varieties and their application in functional foods.

## 1. Introduction

Sorghum is an important cereal grain widely cultivated in the world due to its drought resistance, which is regarded as animal feed in Western countries and as a staple food crop cultivated across Africa’s arid regions [1]. In recent years, researchers have shown a surge of interest in health-promoting constituents—such as resistant starch (RS), polyphenols, and dietary fiber—along with rising consumer demand for functional grain ingredients. Sorghum is a rich source of RS, peptides, phenolics and dietary fiber, and has potential benefits for health, especially in regulating energy intake and metabolic health, which has drawn attention. In addition, sorghum proteins are non-allergenic, making it safe for patients with celiac disease [2,3], positioning sorghum as a viable ingredient for developing specialized foods [4]. Whole sorghum flour (WSF) is used as a wheat substitute to make gluten-free staple foods such as bread, biscuits, and noodles and can also enhance their nutritional value [5,6,7]. Existing research has shown that adding whole sorghum flour to food systems is typically associated with a lower digestion rate and glycemic response [8]. However, WSF as a high-proportion substitute for wheat flour results in poor dough viscoelasticity and diminished increased textural roughness. Moreover, the reduction in the anti-digestive properties of the wheat-based product achieved by the higher WSF content was limited. Currently, heat treatment (such as steam explosion and heat–moisture treatment) is commonly used to enhance the anti-digestive properties of sorghum [7,9]; however, these treatments are often accompanied by structural changes such as starch gelatinization and protein denaturation, which in turn affect the quality of the final product.

Therefore, investigation of staple food-making sorghum cultivar resources is an effective measure for sorghum precision breeding and precise food raw material control strategies; such strategies align with the growing consumer preference for “clean-label” products and the requirement of environmentally friendliness. Genotypic differences in sorghum cultivars are associated with their chemical composition and functional properties. Studies have established that darker-pigmented sorghum cultivars typically contain higher levels of tannins and flavonoids, and are associated with strong antioxidant capacity [10,11]. Sorghum from waxy, non-waxy and partial-waxy genotypes differs markedly in starch structure; thus, it exhibits different physicochemical properties and digestive characteristics [3,12,13]. Non-starch components in sorghum cultivars, such as dietary fibers, polyphenols, and proteins, are often believed to be related to sorghum’s anti-digestive properties. The differences in protein content and composition between hard and soft endosperm sorghum promote the encapsulation of starch granules by kafirin proteins through hydrophobic interactions. These interactions slow down the digestion rate of starch by restricting the contact of enzymes [3,5,14]. Polyphenols have been reported to be capable of inhibiting the activity of digestive enzymes or forming complexes with starch, thereby affecting its digestibility. However, the mode of action remains controversial [15,16]. Soluble dietary fibers may regulate the digestion behavior of starch by altering the viscosity of the system and the distribution of water [17,18]. Insoluble dietary fiber, on the other hand, may affect the contact between enzymes and substrates through a physical barrier effect [19]. However, the above-mentioned mechanisms are mostly based on indirect evidence or model predictions, and their actual roles in complex food systems still lack systematic verification. The indigestibility of sorghum starch (SS) is generally considered to be related to its composition and structural characteristics, but the relationship between these structural differences among different varieties and their digestive behaviors has not been fully elucidated.

Hence, this study was undertaken to systematically investigate the influence of the chemical composition and starch structure of eight hybrid sorghum cultivars developed by this institution and their digestion characteristics. The key point lies in clarifying the correlation mechanism between multi-component and starch digestibility, especially the relationship between the multi-scale structural analysis (e.g., the ratio of amylose to amylopectin, thermal and pasting properties, long-range and short-range ordered structure, lamellar structure and molecular weight analysis) and starch digestibility. It should be noted that this study mainly relies on correlation analysis to explore the potential structure–function relationship rather than establishing a clear causal mechanism. The results of this study can provide basic data for understanding the relationship between different components of sorghum and their digestive characteristics, and offer references for subsequent mechanism-based research and the development of functional sorghum products.

## 2. Materials and Methods

### 2.1. Materials

Eight sorghum cultivars were selected as experimental materials: AH-1, AH-2, AH-3, AH-4, AH-5, AH-6, AH-7, and AH-8. All materials were cultivated at the experimental base of the College of Agriculture, Anhui Science and Technology University (Fengyang, China), throughout their entire growth period, and uniformly harvested at physiological maturity [20]. The primary biochemical reagents used included α-amylase (200 U/mg) and starch glucosidase (10 U/mg), purchased from Yuan Ye (Shanghai, China), and 3,5-dinitrosalicylic acid (DNS), purchased from Maclin (Shanghai, China). All other reagents were of analytical grade.

### 2.2. Determination of Physicochemical Properties of Sorghum Flour

#### 2.2.1. Basic Components Analysis

In accordance with the Chinese National Standards, the moisture [21], ash [22], protein [23], lipids [24] and total dietary fiber [25] of 8 sorghum cultivars were determined.

#### 2.2.2. Free Phenolic Contents Analysis

The free phenol content was determined according to the method described by Yu et al. [26] with slight modifications. Briefly, the 8 sorghum cultivars were freeze-dried, ground into a sorghum flour, and sieved through an 80-mesh screen. Subsequently, 0.2 g of the sorghum flour sample was accurately weighed and extracted with 4 mL of preheated (70 °C, 30 min) 70% (*v*/*v*) methanol aqueous solution for 10 min under shaking. Then, the solution was centrifuged (6000 rpm, 10 min, 4 °C). The extraction was performed twice. The combined supernatants underwent dilution to 10 mL with 70% methanol.

For polyphenol quantification, 1 mL of the extract was mixed with 4.5 mL of Folin–Ciocalteu reagent and allowed to react in the dark at room temperature for 3 min. Then, 5 mL of 7.5% (*w*/*v*) sodium carbonate (Na_2_CO_3_) solution was added, and the mixture was further incubated in the dark for 60 min. The absorbance was measured at 765 nm using an Ultraviolet–visible spectrophotometer. A standard curve was plotted using gallic acid as the standard to determine the polyphenol content.

#### 2.2.3. Tannin Content Analysis

The tannin content was quantified by spectrophotometry, following the method described by Palacios et al. [27] with minor modifications. Briefly, 1.00 g of 8 distinct sorghum flour cultivars was subjected to methanol extraction (10 mL) at 25 °C for 12 h under continuous agitation. Following centrifugation at 3000 rpm for 10 min, the supernatant was carefully decanted. This extraction procedure was repeated twice, and the combined supernatants were brought to a final volume of 25 mL with methanol. For colorimetric analysis, 1.00 mL of the methanolic extract was reacted with 5 mL of freshly prepared vanillin-HCl reagent (consisting of 4% *w*/*v* vanillin in methanol and 8% *v*/*v* concentrated HCl in methanol at 1:1 ratio). The reaction mixture was incubated at room temperature in the dark for 20 min. Subsequently, the absorbance was determined at a wavelength of 500 nm using a UV-visible spectrophotometer. Quantification was performed based on a standard calibration curve constructed using catechin (0~250 μg/mL).

### 2.3. Isolation of Sorghum Starch

Sorghum starch (SS) was isolated from 8 sorghum cultivars by an alkaline steeping procedure from Ma et al. [28]. The sorghum grains were subjected to dry sieving for cleaning residues. Cleaned grains were immersed in a 0.2% NaOH solution at a ratio of 1:10 (*w*/*v*) and placed in a 40 °C water bath for 2.5 h. This step can facilitate seed coat removal. After steeping, the sorghum grains were rinsed exhaustively with distilled water until the wash water reached a neutral pH (7.0). The debranched sorghum grains were homogenized in distilled water at a 1:5 (*w*/*v*) ratio using a high-speed tissue homogenizer. The homogenate was then filtered through a 120-mesh sieve. After sedimentation, the upper supernatant was discarded. The sediment was centrifuged (10,000 rpm, 15 min) to remove the protein-rich supernatant. This purification process was repeated until a clear supernatant and a pure white starch sediment were obtained. Finally, the purified starch was evenly distributed in a glass container and dried in an oven at 40 °C for 24 h. The dried starch was then ground and sieved through a 100-mesh screen. The obtained fine starch was stored at 4 °C prior to analysis.

### 2.4. Starch Composition Analysis

#### 2.4.1. Total Starch

The total starch (TS) content of 8 distinct sorghum flour cultivars was quantified following the enzymatic method described by Zhang et al. [29] with modifications. Briefly, 100 mg of freeze-dried sorghum flour (80 mesh) was suspended in 5 mL of distilled water and gelatinized in a boiling water bath for 30 min. After cooling to room temperature, 6 mL of 2 M KOH solution was added, and the mixture was magnetically stirred for 30 min to ensure complete dispersion. Then, 3 mL of sodium acetate buffer (0.2 M, pH 5.2) was added to the mixture, and the pH of the mixture was adjusted to 5.2 with 2 M HCl or 0.5 M NaOH as needed. The sample was incubated with amyloglucosidase (600 U) in a shaking water bath (55 °C, 120 rpm) for 45 min to hydrolyze starch into glucose. After centrifugation at 3000× *g* for 10 min, the supernatant was collected. The glucose content in the supernatant was quantified via the 3,5-dinitrosalicylic acid (DNS) method.

For isolated SS, 100 mg of the 8 distinct SS cultivars was uniformly mixed with 10 mL of sodium acetate buffer (0.2 M, pH 5.2) and heated at 95 °C for 20 min to achieve complete gelatinization. The sample was then subjected to enzymatic hydrolysis under identical conditions (amyloglucosidase: 600 U; 55 °C, 120 rpm, 45 min), followed by centrifugation (3000 rpm, 10 min) and DNS-based glucose quantification.

#### 2.4.2. Amylose Content

The amylose content was quantified according to the iodine-binding method adapted from the Chinese National Standard [30]. First, 100.0 mg of the 8 distinct sorghum flour cultivars was initially moistened with 1 mL of anhydrous ethanol, then solubilized in 9 mL NaOH (1 mol·L^−1^) using a boiling water bath for 10 min. Immediately after, the solution was cooled in an ice bath, and diluted with distilled water to 100 mL. Lipid removal was achieved by triple extraction of 20 mL aliquots with petroleum ether (3 × 10 mL) involving 10 min vortexing and 15 min standing for phase separation. The defatted aqueous phase (5 mL) was then reacted with 50 mL of distilled water, 1.00 mL acetic acid (1 mol·L^−1^), and 1.00 mL iodine reagent in a 100 mL volumetric flask. Following 10 min of dark incubation, the absorbance of the mixture was measured at 620 nm using a UV-Vis spectrophotometer. A calibration curve was established using potato amylose standards (0–2.00 mg) in the presence of 2 g KI and 0.2 g iodine reagent. The amylose content (AC) was calculated by referencing the standard curve established using potato amylose standards under the same experimental conditions. The amylopectin content (ATC) was calculated by subtracting the amylose content from the total starch content. Among them, the ratio of amylopectin to amylose (ATA) is the content of amylopectin divided by the content of amylose; the proportion of amylopectin (ATR) is the content of amylopectin divided by the total starch content.

### 2.5. In Vitro Starch Digestion

In vitro starch digestion calculations were performed for the 8 distinct sorghum flour cultivars and their isolated starches were analyzed according to [31,32]. Briefly, 100.0 mg of WSF or SS was accurately weighed into a 50 mL conical flask and gelatinized in 15 mL of 0.2 M sodium acetate buffer (pH 5.2) using a boiling water bath with continuous stirring for 30 min. After cooling to room temperature, the solution was equilibrated at 37 °C for 10 min in a temperature-controlled water bath shaker. Enzymatic hydrolysis was initiated by adding 10 mL of enzyme solution (290 U/mL porcine pancreatic α-amylase and 3000 U/mL amyloglucosidase in acetate buffer), followed by incubation at 37 °C with continuous shaking (150 rpm). Aliquots (0.4 mL) were withdrawn at 0, 20, 30, 60, 90, 120, 150, and 180 min intervals and immediately mixed with 2 mL of anhydrous ethanol to terminate the reaction. The glucose concentration in each aliquot was determined against a glucose standard curve using the DNS method. The starch content in each fraction was then calculated as follows:

$$RDS(\%)=\frac{G_{20}-G_{0}}{TS}\times 0.9\times 100$$

$$SDS(\%)=\frac{G_{120}-G_{20}}{TS}\times 0.9\times 100$$

$$RS\left(\%\right)=100-(RDS+SDS)$$

Among them, G_20_ represents the glucose released within the first 20 min, G_120_ represents the glucose released within the first 120 min, and TS stands for the total starch content.

The kinetics of sample starch hydrolysis were described by the nonlinear first-order rate equation (C_t_ = C_∞_(1 − e^−kt^)) proposed by Liu et al. [33], where C_t_ represents the starch hydrolysis extent at any digestion time t (min), C_∞_ represents the maximum starch hydrolysis extent at equilibrium, and k (min^−1^) denotes the first-order kinetic coefficient. The hydrolysis index (HI) is the ratio of the area under the hydrolysis curve (AUHC) for each sample to fresh white bread’s AUHC. The estimated glycemic index (eGI) was calculated according to the following formula:

eGI=0.862HI+8.198

### 2.6. Microscopic and Multi-Scale Structural Analysis

#### 2.6.1. Scanning Electron Microscopy (SEM)

The granule morphology of the 8 distinct SS cultivars was examined by scanning electron microscopy (ZEISS Sigma 300, Oberkochen, Germany). Prior to analysis, SS samples were evenly dispersed on conductive tape and sputter-coated with gold using an ion sputtering coater to ensure adequate surface conductivity. SEM images were captured at magnifications of 300× and 2000× under high-vacuum mode using an accelerating voltage of 10 kV.

#### 2.6.2. Fourier Transform Infrared Spectroscopy (FTIR)

The FTIR spectra of the 8 distinct SS cultivars was conducted using an FTIR-850 FTIR spectrophotometer (Tianjin Gangdong Science and Technology Co., Ltd., Tianjin, China). SS sample (1 mg) was accurately weighed and triturated with KBr in a 1:100 (*w*/*w*) ratio using an agate mortar. The mixture was subsequently compressed under vacuum into a transparent pellet (1 mm thickness) employing a hydraulic pellet press. Afterward, the absorption spectrum was measured over the 4000–400 cm^−1^ range with 32 scans at a resolution of 4 cm^−1^.

#### 2.6.3. Molecular Weight Determination

The molecular weight distribution of the 8 distinct SS cultivars was analyzed using an Agilent 1260 Infinity II GPC system (Mark-Houwink Alpha Concentration Detector, Beijing Polytec Instruments Co., Ltd, Beijing, China) following a modified protocol based on Bai et al. [34]. Starch sample (100 mg) was dissolved in 10 mL of dimethyl sulfoxide (DMSO) containing 0.5% (*w*/*v*) lithium bromide (LiBr) under continuous magnetic stirring at 90 °C for 24 h to ensure complete dissolution. Upon cooling to room temperature, the solutions were subsequently filtered through 0.22 μm organic phase membrane filters to remove insoluble particulates. Chromatographic analysis was performed by injecting 50 µL aliquots into the GPC system equipped with a differential refractive index detector (RID). The separation was achieved using the following optimized parameters: mobile phase DMSO with 0.5% LiBr; flow rate 1 mL/min; detection wavelength 658 nm; column temperature 50 °C; run time 60 min. The system was calibrated with dextran standards of known molecular weights (342 to 2.350 × 10^6^ kDa) to establish the calibration curve. Data acquisition and analysis were carried out via the Agilent GPC software (Agilent GPC/SEC Software A.02.02 [281], Agilent Technologies, Beijing, China).

#### 2.6.4. X-Ray Diffraction (XRD)

Crystalline structure analysis of the 8 distinct SS cultivars was performed using an X-ray diffractometer (Ultima IV, Rigaku, Tokyo, Japan). The instrument was configured with a copper Kα radiation source (λ = 0.154 nm) operating at 35 kV and 25 mA. Scans were conducted in continuous mode over a 2θ range of 2–35° with a scanning rate of 1°/min. The relative crystallinity (RC) of the starch samples was estimated by the peak area method [34]:

$$Relative\;crystallinity(\%)=\frac{Area\;of\;peaks}{Total\;Area}\times 100$$

The peak area represents the sum of the crystalline peak areas (area of crystalline peaks = total area − non-crystalline area), while the total area denotes the sum of all peak areas.

#### 2.6.5. Small-Angle X-Ray Scattering (SAXS)

The nanostructural characterization of the 8 distinct SS cultivars was performed following an adapted protocol based on Cai et al. [35]. Approximately 1 g of SS sample was precisely weighed and transferred to a 10 mL centrifuge tube, followed by suspension in 5 mL of deionized water. The suspension was vortex-mixed to ensure complete dispersion without particle aggregation, followed by centrifugation at 6000× *g* for 5 min. The supernatant was carefully decanted, and the precipitate was collected for SAXS analysis. For SAXS measurements (Xenocs—Soleil SWING system), the prepared sample was loaded into a liquid sample cell and sealed with Kapton film to eliminate air bubbles. After 5 min equilibration at room temperature, measurements were conducted using the following conditions: radiation source Cu-Kα (λ = 0.154 nm), voltage 50 kV, current 0.6 mA, and exposure time 20 min. The SAXS experimental data were systematically analyzed using SAXA analysis software (fit2D) to determine key structural parameters of the starch lamellar organization; we obtained the long period of lamellae formed by the starch crystalline region and amorphous region (average distance L between lamellae), the lamellae thickness of the crystalline region (dc) and the amorphous region thickness (da).

#### 2.6.6. Differential Scanning Calorimetry (DSC)

The crystallinity characteristics of the 8 distinct SS cultivars were analyzed using DSC (Model DSC3, Mettler-Toledo, Zurich, Switzerland). SS samples (3.00 ± 0.01 mg) were accurately weighed into a standard aluminum crucible and hydrated with 9 μL of deionized water (1/3 *w*/*v* starch-to-water ratio). The sealed crucible was equilibrated at 4 °C for 12 h. Thermal analysis was performed under a nitrogen atmosphere at a flow rate of 50 mL/min, with an empty crucible serving as the reference. The testing program was heated from 25 °C to 125 °C at a rate of 10 °C/min. The gelatinization endotherm was characterized by onset (T_o_), peak (T_p_), and conclusion temperatures (T_c_), along with the enthalpy change (ΔH).

#### 2.6.7. Pasting Properties

The gelatinization properties of the 8 distinct SS cultivars were evaluated using a Rapid Visco Analyzer (RVA-4800; Perten Instruments, Sydney, Australia). SS suspensions were prepared in RVA aluminum canisters at a concentration of 3.00 g starch per 25.00 g distilled water. The suspensions were homogenized by stirring with a glass rod prior to loading into the RVA. The pasting parameters were as follows: the mixture was maintained at 50 °C for 1 min, and then heated to 95 °C at a rate of 12 °C/min. Following gelatinization, the tested samples were maintained at 95 °C for 2.5 min, cooled to 50 °C in 3.75 min and balanced at 50 °C for 2 min. Viscosity curves of the 8 distinct SS cultivars during pasting were recorded. The key pasting parameters were determined from the viscosity curves, including peak viscosity (PV, cP), trough viscosity (TV, cP), breakdown viscosity (BV, cP), final viscosity (FV, cP), setback viscosity (SV, cP), peak time (PT, min), and pasting temperature (PaT, °C).

### 2.7. Data Analysis

All experimental data were statistically analyzed using Microsoft Excel. Graphical representations were prepared with OriginPro 2024 (OriginLab Corp., Northampton, MA, USA). Significance testing was conducted through one-way ANOVA with Tukey’s post hoc multiple-comparison test (*p* < 0.05) using SPSS Statistics 27.0 (SPSS Inc., New York, NY, USA).

## 3. Results and Discussion

### 3.1. In Vitro Starch Digestion and Estimated Glycemic Index (eGI) Analysis

**Figure 1:**
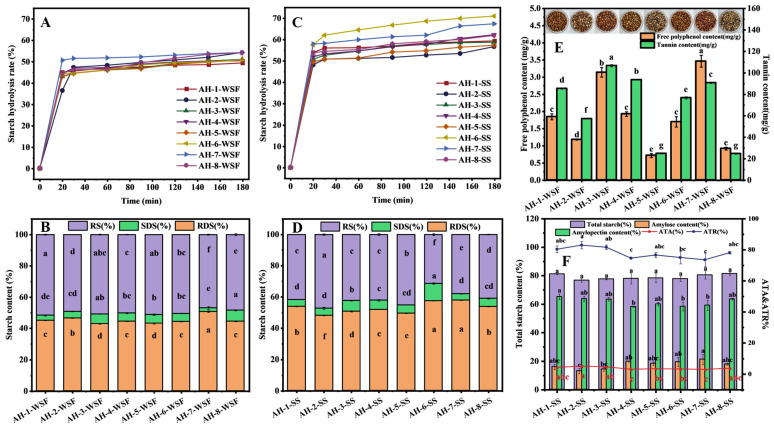
Hydrolysis curves and starch components (RS: resistant starch; SDS: slowly digestible starch; RDS: rapidly digestible starch) of the eight distinct whole sorghum flour (WSF) cultivars (**A**,**B**) and their sorghum starch (SS) (**C**,**D**); free phenolic content and tannin content of the eight distinct WSF cultivars (**E**); and starch composition (total starch (TS), amylose content (AC), amylopectin content (ATC), ATA: amylopectin-to-amylose ratio, ATR: amylopectin ratio) of the eight distinct SSs (**F**). Different lowercase letters indicate significant differences within the group (*p* < 0.05).

As shown in Figure 1A,C, the starch hydrolysis curves of sorghum flour and isolated starch from the eight different sorghum cultivars exhibited distinct differences, with the corresponding starch digestion kinetic parameters listed in Table 1. The equilibrium concentration (C_∞_) reflects the total amount of digestible starch, while the kinetic constant (k) represents the rate of hydrolysis. The results indicated that the starch digestion extent of WSF was lower than that of the corresponding isolated starch in all cultivars. For instance, the C_∞_ of whole flour from cultivar AH-2 was 51.50%, only 1.61% lower than that of its isolated starch, whereas the C_∞_ of whole flour from cultivar AH-6 was 48.80%, 19.41% lower than that of its isolated starch. This reveals that the non-starch components in sorghum significantly influence its anti-digestive properties, and this effect is closely related to differences in the non-starch composition. It is well established that non-starch components including proteins, dietary fiber (DF), polyphenols, and lipids can retard starch digestion [36,37]. The C_∞_ value among the 8 WSF samples varied by only 4.88% and the variation among their isolated starches was substantially larger at 15.1%. The variation in the C_∞_ value depends on inherent differences in different starch cultivars. The hydrolysis index (HI) and estimated glycemic index (eGI) of isolated starch respectively varied by 17.21 and 14.83, whereas that for WSF was only 6.34 and 5.46. The HI and eGI of WSF show lower differences, which could be attributed to the non-starch components. Studies have reported that soluble DF can adsorb polyphenols via non-covalent interactions, thereby mitigating their inhibitory effect on amylase [38,39]; Li et al. [40] reported that proteins interfere with the inhibitory effect of polyphenols on enzymes by prompting the dissociation of polyphenol–enzyme complexes.

The resistant starch (RS) content of the WSF among the eight sorghum cultivars ranged from 46.92% to 51.63%, with a variation of 4.71%, while the RS content of their isolated starches ranged from 31.36% to 47.27%, with a variation of 15.91% (Figure 1B,D). Indeed, Girard et al. [10] also noticed a higher starch digestibility resistance compared to other cereals. The difference in RS content among the eight sorghum cultivars was more significant in isolated starches than in WSF. Significant variation among the isolated starches of cultivars AH-2, AH-5, AH-6, AH-7 and AH-8 was observed in the RS content, reflecting the differences in starch structure across cultivars. The variation in starch composition (including rapidly digestible starch (RDS) content and slowly digestible starch (SDS) content) among isolated starches across different cultivars consistently exceeded that observed in WSF. These results suggested that non-starch components and the composition and structure of starch were related to the anti-digestive properties of sorghum flour.

### 3.2. Compositional Analysis of Sorghum

#### 3.2.1. Non-Starch Components

The eight sorghum cultivars selected for this study had significantly different non-starch components (protein, fat, DF, polyphenol, and tannin contents) and starch composition except for moisture and ash contents (Figure 1E,F and Table 2). When non-starch components were removed from WSF, the differences in eGI among different varieties were significantly reduced. The eGI values of varieties AH-4 and AH-6 only differed by 0.63, while the eGI of cultivar AH-6 was 9.82 higher than that of cultivar AH-4 (Table 1). The results indicated that the differences in the composition of non-starch components among different sorghum varieties corresponded to the variations in their eGI performance. Cultivar AH-6 had a significantly higher total DF content (by 1.62 g/100 g) than cultivar AH-4. The WSF of cultivars AH-4 and AH-7 exhibited higher free-polyphenol and tannin content than that of cultivar AH-6; however, the differences in free polyphenols and tannin content among different varieties did not show a consistent trend. Furthermore, the contents of dietary fiber, protein, free polyphenols and tannins in the WSF of variety AH-3 were all higher than that of variety AH-5. Similar component difference characteristics were also observed between cultivar AH-1 and cultivar AH-3. The eGI value of the WSF from cultivar AH-2 was significantly higher than that of the WSF from cultivar AH-5. Overall, different sorghum varieties show significant differences in non-starch components such as dietary fiber, protein and phenolic substances, and present distinct combination characteristics.

#### 3.2.2. Starch Components

As shown in Figure 1F, total starch content did not differ significantly among the cultivars, but the ratio of amylose to amylopectin varied to some extent, which may be one of the reasons for the significant differences in starch digestibility among the eight sorghum cultivars. In terms of amylose content, cultivar AH-7 had an amylose content of 21.31%, which belongs to the high-amylose category [41]. Cultivar AH-7 also exhibited the highest rapidly digestible starch (RDS) content, reaching 57.94% (Figure 1D). Studies have shown that amylose is more susceptible to hydrolysis by amylase during the early stages of digestion [42]. As hydrolysis continues, hydrolyzed amylose may re-aggregate to form RS [42,43]. Comparing cultivar AH-7 with cultivar AH-6, it was found that under similar amylopectin content, cultivar AH-7 produced more resistant starch in the later stages of digestion, with a higher content than cultivar AH-6. This result is consistent with previous findings that waxy sorghum with lower amylose content shows higher digestibility compared to non-waxy sorghum [14]. Despite cultivar AH-2 and AH-8 having similar total starch and amylopectin content, cultivar AH-2 had lower amylose content and exhibited a significantly lower RDS in the early stage of digestion and a higher RS content. Although this result appears inconsistent with the earlier speculation, it has been documented in previous studies. Sang et al. [12] reported that heterowaxy sorghum had lower amylose content than regular sorghum but higher RS content. There findings indicate that the digestion kinetics of SS are governed by both its composition [35] and, likely, its fine structural features [44,45]. Other studies have also suggested that even if low-amylose rice contains higher amylose content than waxy rice, its weaker crystalline structure may result in lower RS content [43]. Additionally, cultivar AH-2 SS exhibited a higher amylopectin content and amylopectin/amylose ratio, suggesting that its higher RS level may be attributed to both the content and the fine structure of amylopectin. Therefore, to clarify the differences in anti-digestibility among the eight sorghum cultivars, further analysis of the multi-scale structure of starch is required.

### 3.3. Granular Morphology, Thermal Properties, and Multi-Scale Structure Analysis

#### 3.3.1. Observation of Starch Granule Morphology

**Figure 2:**
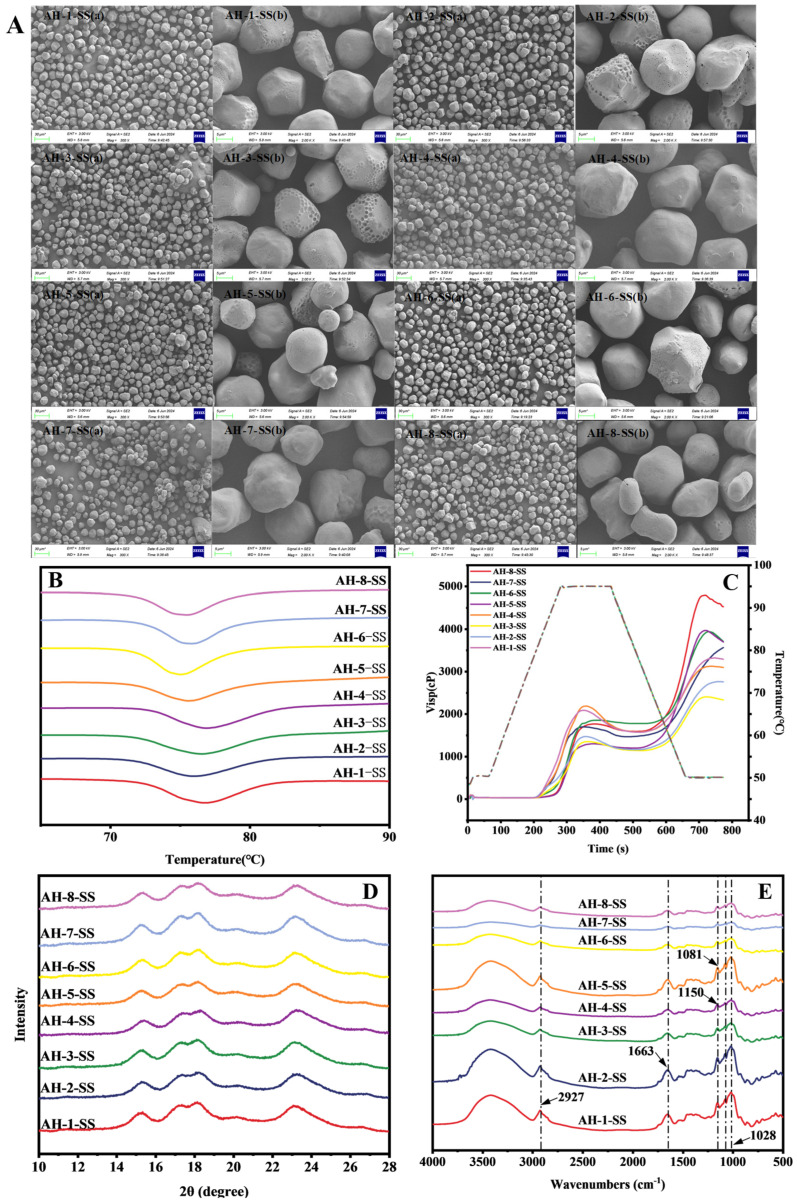
Microscopic morphology (**A**), DSC (**B**), RVA profiles (**C**), XRD (**D**) and FTIR (**E**) of the eight distinct sorghum starch (SS).

As shown in Figure 2A, the granular morphology of all starches from the eight sorghum cultivars displayed irregular polygonal or spherical shapes with smooth surfaces, and no obvious non-starch components were attached, indicating high extraction purity [7]. Micropores and dents were evident on the surfaces of SS granules across the eight sorghum cultivars. Research has demonstrated that micropores provide channels for enzymes to access the interior of starch granules [46], as well as the formation of concavities induced by the interaction between alkali and surface proteins or lipids during the alkaline extraction process [47,48], thereby affecting digestion behavior. The relatively evident surface dents were observed in the starch granules of cultivars AH-2 and AH-3, whereas the starch granules of cultivar AH-4 appeared as relatively smooth surfaces with dense micropores and fewer dents. The more pronounced surface dents of cultivar AH-2 and AH-3 may be attributed to their higher protein or lipid contents (Table 2), which led to more severe surface damage during the extraction process. Cultivar AH-2 and AH-3 starches exhibited higher levels of RS content, revealing that the higher number of dents in starch granules was not correlated with a decrease in RS content (Figure 1D).

#### 3.3.2. Thermal Properties of Sorghum Starch

As shown in Figure 2B and Table 3, significant differences were observed among the eight sorghum cultivars in the T_o_, T_p_, T_c_, and △H. The T_o_, T_p_, and T_c_ values of cultivar AH-4 starch were the highest at 72.64 °C, 76.92 °C, and 82.07 °C, respectively. This may be due to its relatively complete crystalline structure and higher crystallinity [49]. A previous study showed that amylose leaching in cultivar AH-4 starch could restrict the mobility of amylopectin, thereby increasing its gelatinization temperature [50]. Although cultivar AH-7 starch had the highest amylose content (Figure 1F), its gelatinization temperature was relatively low (Table 3). Compared to cultivar AH-7 starch, the T_p_, and T_c_ values of cultivar AH-6 starch were higher. This result is consistent with the report by Cui et al. [51], indicating that when amylose content is below 30%, increasing its concentration can enhance the lamellar structure of crystalline and amorphous regions in the early stage of gelatinization, but further increases ultimately lead to its deterioration. The △H value is strongly associated with the degree of ordered structure [52]. As shown in Table 3, the △H value of cultivar AH-7 starch was higher than that of cultivar AH-6 starch. The higher △H value of cultivar AH-7 starch may be due to its more complete crystalline structure, which is consistent with its higher RS content. Compared to cultivar AH-2 starch, the higher T_o_ value and lower T_c_ value of cultivar AH-8 starch may be attributed to the dissolution of amylose at the initial stage of gelatinization and rapid structural breakdown at later stages. The higher T_c_ value of cultivar AH-2 starch may be due to its longer amylopectin chain length [42]. In previous studies, amylopectin chain length was strongly associated with starch digestibility [53,54]. Indeed, the △H and T_c_ values of cultivar AH-1, AH-3, and AH-4 starches were similar, and their starches exhibited similar resistance to digestion. Cultivar AH-5 starch had a lower △H value but higher RS content, which may be related to the formation of aged starch after starch gelatinization.

#### 3.3.3. Pasting Properties of Sorghum Starch

The pasting properties of starch can reflect its thermal stability, swelling capacity, gel-forming ability, and the integrity of its crystalline structure. The pasting parameters of starches among the eight sorghum cultivars are presented in Figure 2C and Table 4. Cultivar AH-1 and AH-4 starches exhibited higher peak viscosity and breakdown viscosity. The result indicated that the leaching of amylose in cultivar AH-1 and AH-4 starches resulted in structural damage to increase viscosity. Notably, the setback viscosities of cultivar AH-1 and AH-4 starches were comparable, indicating a similar tendency for retrogradation, consistent with their similar RS contents. As shown in Table 4, the peak and trough viscosities of cultivar AH-6 and AH-8 starches were similar, whereas the △H and setback values of cultivar AH-8 starch were higher than those of cultivar AH-6 starch. This phenomenon may be due to the rearrangement of amylose in cultivar AH-8 starch during cooling and the formation of interpenetrating network structure with amylopectin. This aligns with the digestion data (Table 1 and Figure 1D), suggesting that the stronger network structure hinders the contact between amylase and substate and increases the RS content of cultivar AH-8 starch. Cultivar AH-7 starch demonstrated greater gel strength and setback values compared to cultivar AH-2 and AH-3 starches during pasting; however, its RS content was found to be lower. In addition, although the setback viscosity of cultivar AH-7 starch was also higher than that of cultivar AH-5 starch, cultivar AH-7 starch displayed lower T_c_, pasting temperature, and RS content than cultivar AH-5 starch. Cultivar AH-7 starch with the highest amylose content (Figure 1F) exhibited the lowest pasting stability. These findings suggest that amylose readily leaches out during heating due to its weaker binding forces, thereby reducing the thermal stability of cultivar AH-7 starch [55]. Although cultivar AH-2 and AH-3 starches have more surface dents (Figure 2A), their stability during heating was better. These results suggest that the higher RS content is likely primarily governed by their fine structure of amylopectin, such as its ordered arrangement, lamellar organization, and chain-length distribution.

#### 3.3.4. Long-Range Crystalline and Short-Range Ordered Structures of Sorghum Starch

To further investigate the structural differences in starches among the eight sorghum cultivars, the long-range ordered structure of starch was performed using X-ray diffraction (XRD), with results shown in Figure 2D and Table 3. Cultivar AH-2 and AH-3 starches showed the highest relative crystallinity (RC), while cultivar AH-6 starch showed the lowest; cultivar AH-1, AH-4, and AH-7 starches had similar crystallinity levels. The results indicated that despite cultivar AH-2 and AH-3 starches exhibiting more surface dents, they possessed a more complete long-range ordered structure. This may explain the superior anti-digestibility of cultivar AH-2 and AH-3 starches. Additionally, the short-range ordered structure of starches among the eight sorghum cultivars was analyzed using Fourier transform infrared spectroscopy (FTIR) (Figure 2E and Table 3). The degree of short-range order is presented in the form of DO value (1047/1022 cm^−1^), and the compactness of double-helical structures is the DD value (995/1022 cm^−1^) [34]. Jiang et al. [56] reported that short-range ordered structure and double-helix density were highly associated with the anti-digestibility of starch. Cultivar AH-5 starch has the highest DD value, which could be attributed to the higher amylose content and increased entanglements between amylose chains in forming a double-helix structure [57]. Cultivar AH-5 starch has the lowest DO value, which indicates that its short-range ordered structure is poor. Cultivar AH-2 and AH-3 starches, with lower amylose, displayed higher DD values and lower DO values. These results align with the RS content and RC (Figure 1D and Table 3), suggesting that long-range ordered structure and double-helix structure play a crucial role in the anti-digestibility. Although cultivar AH-7 starch showed higher DO values than cultivar AH-2 starch, its DD values and RC were lower than cultivar AH-2 starch. The cultivar AH-7 starch showed the lowest RS content, which is consistent with the prior suggestion. In addition, despite cultivar AH-1, AH-4, and AH-6 starches showing comparable DO values, the RS content of cultivar AH-1 and AH-4 starches was significantly higher than that of cultivar AH-6 starch. This result could be due to the higher RC of cultivar AH-1 and AH-4 starches.

#### 3.3.5. Lamellar Structure of Sorghum Starch

**Figure 3:**
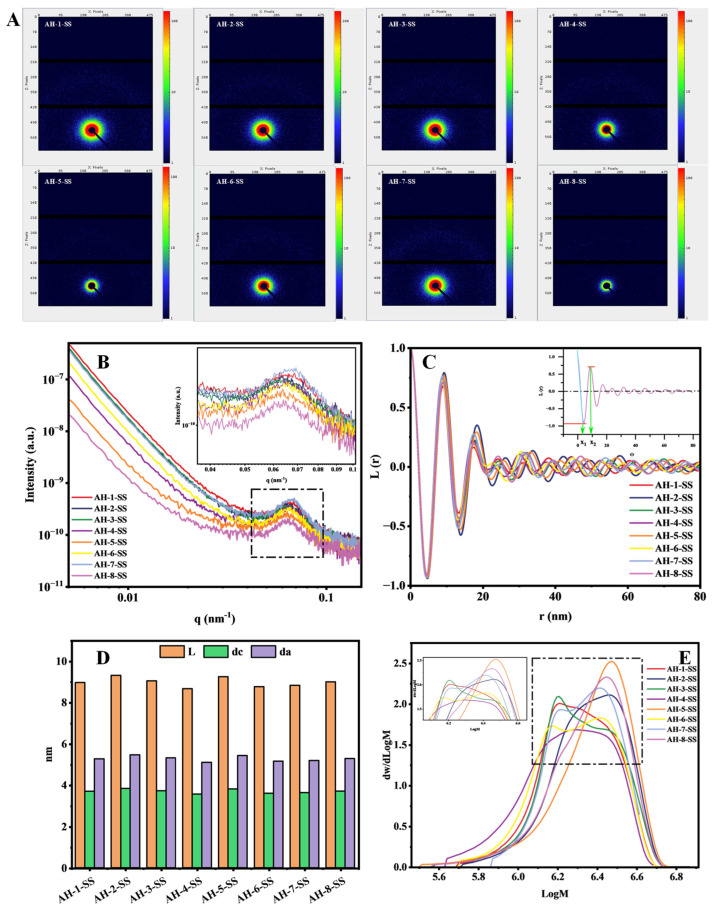
SAXS analysis ((**A**): two-dimensional scattering image; (**B**): one-dimensional scattering curve; (**C**): one-dimensional correlation function curve; (**D**): structural characteristic parameters of starch) and (**E**) GPC profiles of the eight distinct sorghum starch (SS).

Small-angle X-ray scattering (SAXS) has been used to study the nanoscale ordered structure of starches among the eight sorghum cultivars. Chi et al. [58] reported that the scattering peak intensity is primarily determined by the amount of ordered semi-crystalline structures and the electron difference between crystalline and amorphous regions. As shown in Figure 3, it is seen that there were significant differences in SAXS intensity of starches among the eight sorghum cultivars. Cultivar AH-1 starch exhibited a significantly stronger scattering peak intensity than cultivar AH-8 starch. Research indicates that when the amylose content is higher, the contrast in electron density between ordered and amorphous regions is typically enhanced, resulting in sharper scattering peaks [57,59,60]. However, the cultivar AH-1 and AH-8 starches have comparable starch compositions (Figure 1F). Combined with the XRD and FTIR data, it can be inferred that the short-range ordered structures of cultivar AH-1 starch may be more perfect, resulting in its higher anti-digestibility. Although cultivar AH-2 and AH-3 starches have lower amylose contents, both still exhibited high and similar scattering intensities. Their lamellar period thickness (L) and crystalline layer thickness (d_c_) were relatively high, suggesting an overall more compact architecture. From their higher RC and short-range order parameters, it can be inferred that the ordered regions within the starch structure of the two cultivars are tightly arranged. The compact arrangement of cultivar AH-2 and AH-3 starches was related to the higher RS content. The scattering intensity of cultivar AH-5 starch showed a more gradual decrease, which may be due to its relatively loose nanostructure with a larger amorphous layer thickness (d_a_). Combined with the pasting properties (e.g., higher final viscosity and setback value) of cultivar AH-5 starch, the increased anti-digestive properties of cultivar AH-5 starch may be attributed to its more complete gelatinization and the formation of an ordered recrystallized structure.

#### 3.3.6. Molecular Weight Analysis of Sorghum Starch

The results of weight-average molecular weight (M_w_), number-average molecular weight (M_n_), and molecular weight distribution uniformity (polydispersity index, PDI) of starches among the eight sorghum cultivars differed significantly and are presented in Table 3. M_w_ and M_n_ values of starches in cultivars AH-2 and AH-5 are significantly higher than other cultivars. Shi et al. [61] reported that starches with higher M_w_ display better stability during food processing, which can contribute to anti-digestion. This result is consistent with the higher anti-digestibility of cultivars AH-2 and AH-5. Cultivar AH-4 starch exhibited lower M_w_ and M_n_ values and higher PDI, indicating smaller molecular sizes and a broader distribution. Combined with the lower RC and DD content of cultivar AH-4 starch, it can be inferred that the amylopectin of this cultivar has a lower molecular weight and poorer structural order. This molecular structure may be related to its weaker thermal stability during pasting (as reflected by a higher breakdown viscosity) and its lower pasting temperature.

### 3.4. Correlation Analysis Among Parameters

**Figure 4:**
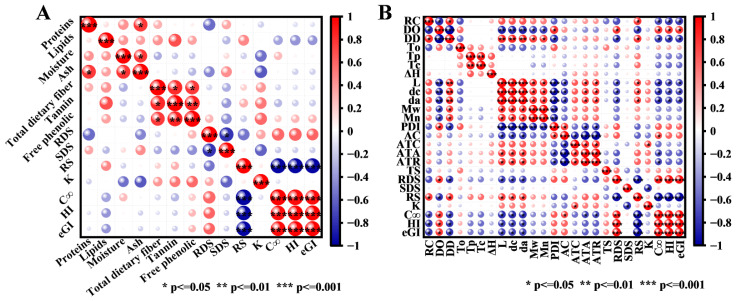
Spearman correlation analysis of physicochemical properties, digestive characteristics, and starch structural features among sorghum varieties ((**A**): Correlation analysis of non-starch components with whole sorghum flour digestive characteristics; (**B**): Correlation analysis of starch structural features with sorghum starch digestive characteristics).

To further elucidate the key components responsible for the anti-digestibility of sorghum, Spearman’s correlation analysis was employed. This analysis investigated the correlation of non-starch components with WSF digestibility (Figure 4A), as well as the correlation of starch components (composition and structure) with SS digestibility (Figure 4B). The correlation analysis revealed no significant correlation between the digestibility parameters of WSF and the content of non-starch components (such as proteins, lipids, ash, total dietary fiber, tannins, and free phenolics). However, there was a significant difference in the digestive properties between WSF and SS (Figure 1A–D). These results suggest interactions among non-starch components, which weaken the correlation between their content and the digestibility of WSF. Notably, a significant positive correlation was found between RC and DD as well as RS, whereas a significant negative correlation existed between RC and DO. These correlation patterns support the earlier inference that long-range ordered structure plays a dominant role in determining anti-digestibility. Additionally, the lamellar parameters (L, d_a_, d_c_) were positively correlated with RS content and negatively correlated with RDS, C_∞_, HI and eGI. The correlations found in this result are in agreement with the above findings (Section 3.3.5). It has been documented that a denser lamellar structure and greater layer thickness enhance the physical barriers to enzyme access. A strong positive correlation was found between M_w_, M_n_ and lamellar parameters (L, d_a_, d_c_) of starches. This correlation is in consensus with the work of Shi et al. [61], which also reports that higher M_w_ is conducive to slow digestion of starch. The PDI value displayed a negative correlation with the DD value, suggesting that starches with lower PDI value exhibit simpler and more homogeneous molecular chains, thereby increasing the DD value [33].

## 4. Conclusions

This study systematically compared the digestive property differences of WSF and isolated SS from eight sorghum cultivars. The starch digestion rate of WSF among the eight sorghum cultivars was slower than that of SS, suggesting that the presence of non-starch components was associated with lower digestibility. Based on the correlation analysis results, there was a certain correlation between the interaction of non-starch components and the digestion behavior of WSF, but the specific mode of action was not clear. In addition, the difference in the anti-digestibility of SS among different varieties was more significant than that of WSF, indicating that there was a correlation between the structural characteristics of starch and its digestion performance. The increase in surface depressions of starch granules in varieties AH-2 and AH-3 did not show a significant correlation with their anti-digestibility. Further correlation analysis results showed that longer amylopectin chain length and higher long-range order (RC) were positively correlated with higher resistant starch (RS) content; cultivars AH-2 and AH-3 had higher DD values and RC, and their RS content was also relatively high, but the above relationship only reflected statistical correlation and could not be used to infer causality. Analysis of the structure of cultivar AH-8 starch revealed that the rearrangement of amylose and the formation of strong network structure are conducive to the increase in RS content. Moreover, the lamellar parameters (L, d_a_, d_c_) of cultivar AH-2 and AH-3 starches was higher, suggesting that a dense and thick lamellar structure effectively hinders enzyme penetration and hydrolysis. This study revealed the statistical correlation laws between different components and starch structural characteristics of sorghum and their digestion behavior, but these results were mainly based on correlation analysis and lacked direct mechanistic verification, and thus a clear causal relationship could not be established. The structural–functional relationship and its mechanism of action need to be further clarified through controlled-variable experiments and structural regulation studies. The results of this study can provide a reference for subsequent mechanism research and the screening of anti-digestible sorghum raw materials.

## Figures and Tables

**Table 1:** Kinetic parameters of the eight distinct whole sorghum flour (WSF) cultivars and their sorghum starch (SS) digestion and glycemic index value.

Sorghum Variety	Kinetics Parameters			HI	eGI
	C_∞_ (%)	K (min^−1^)	R^2^		
AH-1-WSF	48.04 ± 0.17 ^f^	0.13 ± 0.00 ^b^	0.9968	57.84 ± 0.20 ^ef^	58.05 ± 0.17 ^ef^
AH-2-WSF	51.50 ± 0.21 ^b^	0.07 ± 0.00 ^f^	0.9880	59.67 ± 0.23 ^c^	59.64 ± 0.20 ^c^
AH-3-WSF	48.67 ± 0.21 ^e^	0.10 ± 0.00 ^de^	0.9899	57.89 ± 0.24 ^ef^	58.10 ± 0.20 ^ef^
AH-4-WSF	49.28 ± 0.26 ^d^	0.11 ± 0.00 ^c^	0.9964	59.06 ± 0.25 ^d^	59.11 ± 0.21 ^d^
AH-5-WSF	48.34 ± 0.06 ^ef^	0.10 ± 0.00 ^cde^	0.9903	57.61 ± 0.15 ^f^	57.86 ± 0.13 ^f^
AH-6-WSF	48.80 ± 0.18 ^e^	0.11 ± 0.00 ^cd^	0.9910	58.34 ± 0.24 ^e^	58.48 ± 0.21 ^e^
AH-7-WSF	52.92 ± 0.13 ^a^	0.15 ± 0.01 ^a^	0.9978	63.95 ± 0.15 ^a^	63.32 ± 0.13 ^a^
AH-8-WSF	50.98 ± 0.22 ^c^	0.09 ± 0.00 ^e^	0.9810	60.40 ± 0.26 ^b^	60.26 ± 0.23 ^b^
AH-1-SS	57.93 ± 0.07 ^d^	0.13 ± 0.00 ^a^	0.9973	69.72 ± 0.10 ^cd^	68.30 ± 0.08 ^cd^
AH-2-SS	53.11 ± 0.35 ^g^	0.12 ± 0.01 ^b^	0.9921	63.45 ± 0.32 ^g^	62.89 ± 0.23 ^g^
AH-3-SS	57.20 ± 0.16 ^e^	0.10 ± 0.00 ^c^	0.9944	68.24 ± 0.19 ^e^	67.02 ± 0.16 ^e^
AH-4-SS	58.27 ± 0.26 ^cd^	0.10 ± 0.00 ^c^	0.9851	69.26 ± 0.32 ^d^	67.90 ± 0.28 ^d^
AH-5-SS	54.68 ± 0.11 ^f^	0.11 ± 0.00 ^b^	0.9900	65.31 ± 0.10 ^f^	64.49 ± 0.09 ^f^
AH-6-SS	68.21 ± 0.32 ^a^	0.09 ± 0.00 ^d^	0.9929	80.66 ± 0.38 ^a^	77.72 ± 0.33 ^a^
AH-7-SS	63.18 ± 0.16 ^b^	0.11 ± 0.00 ^b^	0.9848	75.45 ± 0.11 ^b^	73.23 ± 0.10 ^b^
AH-8-SS	58.69 ± 0.14 ^c^	0.11 ± 0.00 ^b^	0.9897	70.18 ± 0.12 ^c^	68.69 ± 0.10 ^c^

Note: Values marked with different letters in the same column are significantly different (*p* < 0.05). C_∞_: equilibrium concentration; K: first-order kinetic coefficient; R^2^: The correlation coefficient of the fitted curve; HI: hydrolysis index; eGI: estimated glycemic index.

**Table 2:** Physicochemical properties of the eight distinct sorghum flour cultivars.

Sorghum Variety	Moisture (g/100 g)	Ash (g/100 g)	Proteins (g/100 g)	Lipids (g/100 g)	Total Dietary Fiber (g/100 g)
AH-1-WSF	10.60 ± 0.26 ^c^	0.01 ± 0.00 ^c^	9.47 ± 0.05 ^d^	4.88 ± 0.36 ^a^	9.65 ± 0.01 ^d^
AH-2-WSF	11.63 ± 0.12 ^a^	0.02 ± 0.00 ^bc^	10.59 ± 0.13 ^c^	4.78 ± 0.76 ^a^	7.60 ± 0.02 ^f^
AH-3-WSF	11.08 ± 0.23 ^b^	0.02 ± 0.00 ^a^	13.55 ± 0.12 ^a^	4.90 ± 0.41 ^a^	12.21 ± 0.00 ^a^
AH-4-WSF	10.75 ± 0.12 ^bc^	0.01 ± 0.00 ^c^	10.31 ± 0.05 ^c^	3.80 ± 1.32 ^ab^	9.01 ± 0.00 ^e^
AH-5-WSF	11.14 ± 0.03 ^b^	0.02 ± 0.00 ^ab^	12.71 ± 0.94 ^b^	4.01 ± 0.33 ^ab^	7.16 ± 0.01 ^g^
AH-6-WSF	10.51 ± 0.04 ^c^	0.02 ± 0.00 ^bc^	10.75 ± 0.14 ^c^	3.35 ± 0.06 ^ab^	10.63 ± 0.01 ^b^
AH-7-WSF	11.04 ± 0.14 ^b^	0.02 ± 0.00 ^bc^	11.20 ± 0.20 ^c^	4.01 ± 0.24 ^ab^	9.73 ± 0.02 ^c^
AH-8-WSF	10.76 ± 0.17 ^bc^	0.02 ± 0.00 ^bc^	10.67 ± 0.07 ^c^	3.02 ± 0.12 ^b^	5.55 ± 0.02 ^h^

Note: Values marked with different letters in the same column are significantly different (*p* < 0.05).

**Table 3:** Relative crystallinity, thermal properties and molecular weight of the eight distinct sorghum starch (SS) cultivars.

Sorghum Variety	AH-1-SS	AH-2-SS	AH-3-SS	AH-4-SS	AH-5-SS	AH-6-SS	AH-7-SS	AH-8-SS
T_o_ (°C)	72.35 ± 0.07 ^ab^	71.52 ± 0.08 ^d^	71.73 ± 0.17 ^cd^	72.64 ± 0.14 ^a^	71.84 ± 0.30 ^cd^	72.02 ± 0.42 ^bcd^	71.72 ± 0.05 ^cd^	72.05 ± 0.42 ^bc^
T_P_ (°C)	76.58 ± 0.35 ^ab^	75.84 ± 0.23 ^d^	76.34 ± 0.23 ^bc^	76.92 ± 0.12 ^a^	75.92 ± 0.35 ^cd^	75.67 ± 0.23 ^d^	75.17 ± 0.00 ^e^	75.67 ± 0.23 ^d^
T_C_ (°C)	81.72 ± 0.07 ^ab^	81.62 ± 0.35 ^ab^	81.04 ± 0.16 ^cd^	82.07 ± 0.31 ^a^	81.40 ± 0.57 ^bc^	81.02 ± 0.24 ^cd^	79.74 ± 0.02 ^e^	80.72 ± 0.04 ^d^
ΔH (J/g)	266.05 ± 9.97 ^ab^	257.35 ± 29.20 ^abc^	281.05 ± 18.17 ^a^	276.10 ± 6.79 ^a^	233.90 ± 5.37 ^c^	239.45 ± 4.88 ^bc^	244.40 ± 17.11 ^bc^	253.50 ± 2.84 ^abc^
RC (%)	39.60 ± 1.36 ^ab^	42.08 ± 0.80 ^ab^	42.78 ± 0.99 ^a^	39.73 ± 1.92 ^ab^	41.38 ± 1.86 ^ab^	37.40 ± 1.64 ^b^	40.21 ± 3.28 ^ab^	39.64 ± 0.57 ^ab^
DO (×10^−2^)	102.96 ± 0.13 ^a^	102.13 ± 0.08 ^c^	102.28 ± 0.12 ^bc^	103.02 ± 0.00 ^a^	101.74 ± 0.01 ^d^	103.23 ± 0.02 ^a^	102.55 ± 0.02 ^b^	102.23 ± 0.16 ^c^
DD (×10^−2^)	96.79 ± 0.14 ^d^	97.71 ± 0.08 ^b^	97.54 ± 0.13 ^b^	96.75 ± 0.00 ^d^	98.13 ± 0.01 ^a^	96.52 ± 0.01 ^d^	97.25 ± 0.02 ^c^	97.59 ± 0.17 ^b^
Mw (×10^6^ g/mol)	2.23 ± 0.06 ^bc^	2.47 ± 0.00 ^a^	2.25 ± 0.01 ^bc^	2.13 ± 0.14 ^c^	2.64 ± 0.05 ^a^	2.17 ± 0.03 ^c^	2.42 ± 0.17 ^ab^	2.50 ± 0.05 ^a^
Mn (×10^6^ g/mol)	1.87 ± 0.05 ^d^	2.07 ± 0.02 ^b^	1.82 ± 0.02 ^de^	1.57 ± 0.01 ^f^	2.18 ± 0.08 ^a^	1.74 ± 0.03 ^e^	1.97 ± 0.00 ^c^	2.05 ± 0.03 ^bc^
PDI (Mw/Mn)	1.19 ± 0.00 ^b^	1.20 ± 0.01 ^b^	1.23 ± 0.02 ^b^	1.36 ± 0.10 ^a^	1.21 ± 0.02 ^b^	1.25 ± 0.04 ^b^	1.23 ± 0.09 ^b^	1.22 ± 0.01 ^b^

Note: Values marked with different letters in the same row are significantly different (*p* < 0.05). RC: relative crystallinity; DO: short-range ordering of starch; (R_1047/1022_); DD: double-helix degree (R_995/1022_); T_o_: onset temperature; T_P_: peak temperature; T_C_: conclusion temperature; ΔH: enthalpy of gelatinization; Mw: weight-average molecular weight; Mn: number-average molecular weight; PDI: polydispersity index.

**Table 4:** Pasting properties of the eight distinct sorghum starch (SS) cultivars.

Sorghum Variety	Peak Viscosity (cP)	Trough Viscosity (cP)	Breakdown Viscosity (cP)	Final Viscosity (cP)	Setback Viscosity (cP)	Peak Time (min)	Pasting Temp. (°C)
AH-1-SS	2087.50 ± 12.02 ^a^	1564.50 ± 3.54 ^b^	523.00 ± 8.49 ^b^	3296.00 ± 14.14 ^d^	1731.50 ± 10.61 ^e^	5.87 ± 0.00 ^d^	81.08 ± 0.53 ^c^
AH-2-SS	1442.00 ± 25.46 ^d^	1136.50 ± 20.51 ^e^	305.50 ± 4.95 ^c^	2722.50 ± 41.72 ^f^	1586.00 ± 21.21 ^f^	6.00 ± 0.00 ^c^	81.20 ± 0.64 ^c^
AH-3-SS	1338.50 ± 3.54 ^e^	1120.50 ± 10.61 ^e^	218.00 ± 7.07 ^de^	2328.00 ± 5.66 ^g^	1207.50 ± 16.26 ^h^	6.04 ± 0.05 ^c^	82.35 ± 0.00 ^c^
AH-4-SS	2145.00 ± 48.08 ^a^	1555.50 ± 16.26 ^b^	589.50 ± 31.82 ^a^	3062.00 ± 41.01 ^e^	1506.50 ± 24.75 ^g^	6.00 ± 0.00 ^c^	81.50 ± 0.07 ^c^
AH-5-SS	1309.50 ± 30.41 ^e^	1206.50 ± 26.16 ^d^	103.00 ± 4.24 ^f^	3733.00 ± 56.57 ^b^	2526.50 ± 30.41 ^b^	6.33 ± 0.00 ^b^	92.03 ± 0.11 ^a^
AH-6-SS	1809.00 ± 50.91 ^b^	1742.00 ± 36.77 ^a^	67.00 ± 14.14 ^f^	3695.00 ± 15.56 ^b^	1953.00 ± 21.21 ^d^	6.40 ± 0.00 ^ab^	89.65 ± 0.07 ^b^
AH-7-SS	1698.50 ± 13.44 ^c^	1463.50 ± 12.02 ^c^	235.00 ± 1.41 ^d^	3582.00 ± 32.53 ^c^	2118.50 ± 20.51 ^c^	5.87 ± 0.00 ^d^	81.53 ± 0.04 ^c^
AH-8-SS	1747.00 ± 15.56 ^bc^	1567.00 ± 21.21 ^b^	180.00 ± 5.66 ^e^	4534.50 ± 13.44 ^a^	2967.50 ± 34.65 ^a^	6.44 ± 0.05 ^a^	91.68 ± 0.60 ^a^

Note: Values marked with different letters in the same column are significantly different (*p* < 0.05).

## Data Availability

The original contributions presented in the study are included in the article, further inquiries can be directed to the corresponding author.

## References

[1] Xiong Y., Zhang P.Z., Warner R.D., Fang Z.X. (2019). Sorghum Grain: From Genotype, Nutrition, and Phenolic Profile to Its Health Benefits and Food Applications. Compr. Rev. Food Sci. Food Saf..

[2] Deore A., Athmaselvi K.A., Venkatachalapathy N. (2023). Effect of Ultrasound and Microwave Pretreatment on Sprouting, GABA, Bioactive Compounds, and Other Physicochemical Properties of Sorghum. Grain Oil Sci. Technol..

[3] Semwal J., Meera M.S. (2021). Infrared Modification of Sorghum to Produce a Low Digestible Grain Fraction. J. Cereal Sci..

[4] Awika J.M., Duodu K.G. (2017). Bioactive Polyphenols and Peptides in Cowpea (Vigna Unguiculata) and Their Health Promoting Properties: A Review. J. Funct. Foods.

[5] Yousif A., Nhepera D., Johnson S. (2012). Influence of Sorghum Flour Addition on Flat Bread In Vitro Starch Digestibility, Antioxidant Capacity and Consumer Acceptability. Food Chem..

[6] Espinosa-Ramírez J., Serna-Saldívar S.O. (2016). Functionality and Characterization of Kafirin-Rich Protein Extracts from Different Whole and Decorticated Sorghum Genotypes. J. Cereal Sci..

[7] Ma X.L., Wang H.M., Zhang Y., Li P.Y., Li D.Y., Yu K. (2025). Impact of Steam Explosion on Sorghum Starch Digestibility and Physicochemical Properties. Food Res. Int..

[8] Haziman M.L., Ishaq M.I., Qonit M.A.H., Lestari E.G., Susilawati P.N., Widarsih W., Syukur C., Herawati H., Arief R., Santosa B. (2025). Sorghum Starch Review: Structural Properties, Interactions with Proteins and Polyphenols, and Modification of Physicochemical Properties. Food Chem..

[9] Sun Q., Han Z., Wang L., Xiong L. (2014). Physicochemical Differences between Sorghum Starch and Sorghum Flour Modified by Heat-Moisture Treatment. Food Chem..

[10] Girard A.L., Awika J.M. (2018). Sorghum Polyphenols and Other Bioactive Components as Functional and Health Promoting Food Ingredients. J. Cereal Sci..

[11] Hullings A.G., Sinha R., Liao L.M., Freedman N.D., Graubard B.I., Loftfield E. (2020). Whole Grain and Dietary Fiber Intake and Risk of Colorectal Cancer in the NIH-AARP Diet and Health Study Cohort. Am. J. Clin. Nutr..

[12] Sang Y., Bean S., Seib P.A., Pedersen J., Shi Y.C. (2008). Structure and Functional Properties of Sorghum Starches Differing in Amylose Content. J. Agric. Food Chem..

[13] Nakajima S., Horiuchi S., Ikehata A., Ogawa Y. (2021). Determination of Starch Crystallinity with the Fourier-Transform Terahertz Spectrometer. Carbohydr. Polym..

[14] Wong J.H., Lau T., Cai N., Singh J., Pedersen J.F., Vensel W.H., Hurkman W.J., Wilson J.D., Lemaux P.G., Buchanan B.B. (2009). Digestibility of Protein and Starch from Sorghum (*Sorghum bicolor*) Is Linked to Biochemical and Structural Features of Grain Endosperm. J. Cereal Sci..

[15] Sudlapa P., Suwannaporn P. (2023). Dual Complexation Using Heat Moisture Treatment and Pre-Gelatinization to Enhance Starch–Phenolic Complex and Control Digestibility. Food Hydrocoll..

[16] Zhu S.N., Li J., Li W.Y., Li S.S., Yang X., Liu X.B., Sun L.J. (2022). Enzymic Catalyzing Affinity to Substrate Affects Inhibitor-Enzyme Binding Interactions: Inhibition Behaviors of EGCG against Starch Digestion by Individual and Co-Existing α-Amylase and Amyloglucosidase. Food Chem..

[17] Tu J.C., Adhikari B., Brennan M.A., Cheng P., Bai W.D., Brennan C.S. (2023). Interactions between Sorghum Starch and Mushroom Polysaccharides and Their Effects on Starch Gelatinization and Digestion. Food Hydrocoll..

[18] Ma X.L., Zhang Y., Chu X.B., Wei L.L., Li P.Y., Yu K. (2025). Effects of Steam Explosion on the Structural, Physicochemical, and Functional Characteristics of Dietary Fiber in Sorghum Grains. LWT.

[19] Li J.Y., Lang W.J., Han S., Wu X.Y., Hao F.W., Zhou Y.R., Du R.P., Song C. (2025). Insights into the Mechanisms and Functional Effects of Insoluble Dietary Fiber Modification: A Review. Foods.

[20] Wang K., Wang L.M., Shen Q., Hu L., Xing Z.C., Wang Y.H., Li J.Q. (2025). Association Analysis and Identification of Candidate Genes for Sorghum Coleoptile Color. Agronomy.

[21] (2016). National Standards for Food Safety, Determination of Moisture in Food.

[22] (2016). National Standards for Food Safety, Determination of Ash in Food.

[23] (2016). National Standards for Food Safety, Determination of Protein in Food.

[24] (2016). National Standards for Food Safety, Determination of Fat in Food.

[25] (2023). National Standards for Food Safety, Determination of Dietary Fiber in Food.

[26] Yu K., Huang X.X., He W., Ma X.L., Wu D., Ding Z.G., Li P.Y., Du C.L. (2023). Evaluation of the Effects of Thermal Processing on Antioxidant Activity and Digestibility of Green Tea Noodles: Based on Polyphenol Stability and Starch Structure. J. Cereal Sci..

[27] Palacios C.E., Nagai A., Torres P., Rodrigues J.A., Salatino A. (2021). Contents of Tannins of Cultivars of Sorghum Cultivated in Brazil, as Determined by Four Quantification Methods. Food Chem..

[28] Ma Z.B., Wang C.Y., Tian Y.Y., Zhao D., Wang J.X., Ke F.L., Zheng J., Su J., Bian M.H., Ma Y. (2025). Investigating the Influence of the Molecular Structure and Physiochemical Properties of Starches from Glutinous and Japonica Sorghum on Light-Flavor Liquor Fermentation. Int. J. Biol. Macromol..

[29] Zhang H.H., Jiang Y.L., Pan J.X., Lv Y.J., Liu J., Zhang S.K., Zhu Y.J. (2018). Effect of Tea Products on the In Vitro Enzymatic Digestibility of Starch. Food Chem..

[30] (1987). National Standards for Food Safety, Determination of Amylose Content in Rice, Maize and Millet Grains.

[31] Englyst H.N., Kingman S.M., Cummings J.H. (1992). Classification and Measurement of Nutritionally Important Starch Fractions. Eur. J. Clin. Nutr..

[32] Wang Y., Guo J., Wang C., Li Y., Bai Z., Luo D., Hu Y., Chen S. (2023). Effects of Konjac Glucomannan and Freezing on Thermal Properties, Rheology, Digestibility and Microstructure of Starch Isolated from Wheat Dough. LWT.

[33] Liu F.Y., Guo X.N., Xing J.J., Zhu K.X. (2020). Effect of Thermal Treatments on in Vitro Starch Digestibility of Sorghum Dried Noodles. Food Funct..

[34] Bai Y.P., Zhou H.M., Guo X.N., Zhu K.X. (2022). Structural Changes and Components’ Interactions Alter the Digestion Property of in-Kernel Starch from Thermally Processed Tibetan Qingke. Food Res. Int..

[35] Cai J.W., Man J.M., Huang J., Liu Q.Q., Wei W.X., Wei C.X. (2015). Relationship between Structure and Functional Properties of Normal Rice Starches with Different Amylose Contents. Carbohydr. Polym..

[36] Qadir N., Wani I.A. (2022). In-Vitro Digestibility of Rice Starch and Factors Regulating Its Digestion Process: A Review. Carbohydr. Polym..

[37] Yang Z.L., Zhang Y.Y., Wu Y.W., Ouyang J. (2023). Factors Influencing the Starch Digestibility of Starchy Foods: A Review. Food Chem..

[38] Sun L., Warren F.J., Gidley M.J. (2019). Natural Products for Glycaemic Control: Polyphenols as Inhibitors of Alpha-Amylase. Trends Food Sci. Technol..

[39] Wang K.L., Li M., Han Q.Y., Fu R., Ni Y.Y. (2021). Inhibition of α-Amylase Activity by Insoluble and Soluble Dietary Fibers from Kiwifruit (*Actinidia deliciosa*). Food Biosci..

[40] Li W.Y., Zhang J.F., Bao X.Y., He J., Cao J., Li C.X., Liu X.B., Sun L.J. (2023). Binding Interactions between Protein and Polyphenol Decreases Inhibitory Activity of the Polyphenol against α-Amylase: A New Insight into the Effect of Dietary Components on Starch-Hydrolyzing Enzyme Inhibition. Food Hydrocoll..

[41] Yang L.P., Li L., Jiang F., Zhang Q.L., Yang F., Wang Y.Y., Zhao Z.Y., Ren Q.F., Wang L. (2024). Structural and Physicochemical Characteristics of Starches from Sorghum Varieties with Varying Amylose Content. Food Sci. Nutr..

[42] Zhu L.J., Liu Q.Q., Wilson J.D., Cu M.H., Shi Y.C. (2011). Digestibility and Physicochemical Properties of Rice (*Oryza sativa* L.) Flours and Starches Differing in Amylose Content. Carbohydr. Polym..

[43] Chi C.D., Li X.X., Huang S.X., Chen L., Zhang Y.P., Li L., Miao S. (2021). Basic Principles in Starch Multi-Scale Structuration to Mitigate Digestibility: A Review. Trends Food Sci. Technol..

[44] Benmoussa M., Moldenhauer K.A.K., Hamaker B.R. (2007). Rice Amylopectin Fine Structure Variability Affects Starch Digestion Properties. J. Agric. Food Chem..

[45] Peng Y., Mao B.G., Zhang C.Q., Shao Y., Wu T.H., Hu L.M., Hu Y.Y., Tang L., Li Y.K., Tang W.B. (2021). Influence of Physicochemical Properties and Starch Fine Structure on the Eating Quality of Hybrid Rice with Similar Apparent Amylose Content. Food Chem..

[46] MacGregor A.W., Balance D.L. (1980). Hydrolysis of Large and Small Starch Granules from Normal and Waxy Barley Cultivars by Alpha-Amylases from Barley Malt. Cereal Chem..

[47] Chen X.Y., Zhu L., Zhang H., Wu G.C., Cheng L.L., Zhang Y.Y. (2025). A Review of Endogenous Non-Starch Components in Cereal Matrix: Spatial Distribution and Mechanisms for Inhibiting Starch Digestion. Crit. Rev. Food Sci. Nutr..

[48] Li W.H., Gao J.M., Wu G.L., Zheng J.M., Ouyang S.H., Luo Q.G., Zhang G.Q. (2016). Physicochemical and Structural Properties of A- and B-Starch Isolated from Normal and Waxy Wheat: Effects of Lipids Removal. Food Hydrocoll..

[49] Xiao Y., Liu H., Wei T., Shen J., Wang M. (2017). Differences in Physicochemical Properties and in Vitro Digestibility between Tartary Buckwheat Flour and Starch Modified by Heat-Moisture Treatment. LWT.

[50] Watcharatewinkul Y., Puttanlek C., Rungsardthong V., Uttapap D. (2009). Pasting Properties of a Heat-Moisture Treated Canna Starch in Relation to Its Structural Characteristics. Carbohydr. Polym..

[51] Cui Y., Liu X.N., Lv Q., Chang J.H., Blennow A., Tian Y., Chen S., Liu X.X., Zhong Y.Y. (2025). In Situ Small-Angle X-Ray Scattering Study of the Gelatinization Mechanism of Maize Starches with Varying Amylose Content. Food Hydrocoll..

[52] Liu H., Fan H.H., Cao R., Blanchard C., Wang M. (2016). Physicochemical Properties and in Vitro Digestibility of Sorghum Starch Altered by High Hydrostatic Pressure. Int. J. Biol. Macromol..

[53] Qiao J.W., Jia M., Niu J.H., Zhang Z., Xing B., Liang Y.Q., Li H., Zhang Y.W., Ren G.X., Qin P.Y. (2024). Amylopectin Chain Length Distributions and Amylose Content Are Determinants of Viscoelasticity and Digestibility Differences in Mung Bean Starch and Proso Millet Starch. Int. J. Biol. Macromol..

[54] Zhu J.H., Liu Q.Q., Gilbert R.G. (2024). The Effects of Chain-Length Distributions on Starch-Related Properties in Waxy Rices. Carbohydr. Polym..

[55] Irondi E.A., Adewuyi A.E., Aroyehun T.M. (2022). Effect of Endogenous Lipids and Proteins on the Antioxidant, in Vitro Starch Digestibility, and Pasting Properties of Sorghum Flour. Front. Nutr..

[56] Jiang J.N., Han W.F., Zhao S.M., Liu Q.X., Lin Q.L., Xiao H.X., Fu X.J., Li J.T., Ren K.Z., Lu H.H. (2024). Comparison of Structural and in Vitro Digestive Properties of Autoclave-Microwave Treated Maize Starch under Different Retrogradation Temperature Conditions. Int. J. Biol. Macromol..

[57] Ji Z.L., Yu L., Liu H.S., Bao X.Y., Wang Y.F., Chen L. (2017). Effect of Pressure with Shear Stress on Gelatinization of Starches with Different Amylose/Amylopectin Ratios. Food Hydrocoll..

[58] Chi C.D., Li X.X., Zhang Y.P., Chen L., Xie F.W., Li L., Bai G.H. (2019). Modulating the in Vitro Digestibility and Predicted Glycemic Index of Rice Starch Gels by Complexation with Gallic Acid. Food Hydrocoll..

[59] Liu X.X., Xiao X.M., Liu P., Yu L., Li M., Zhou S.M., Xie F.W. (2017). Shear Degradation of Corn Starches with Different Amylose Contents. Food Hydrocoll..

[60] Zhu J., Zhang S.Y., Zhang B.J., Qiao D.L., Pu H.Y., Liu S.Y., Li L. (2017). Structural Features and Thermal Property of Propionylated Starches with Different Amylose/Amylopectin Ratio. Int. J. Biol. Macromol..

[61] Shi X., Fan C.M., Pan C.M., Zhang F.L., Hou X.G., Hui M. (2024). Analysis of Differences in Physicochemical Properties of Different Sorghum Varieties and Their Influence on the Selection of Raw Materials for Winemaking. Food Chem. X.

